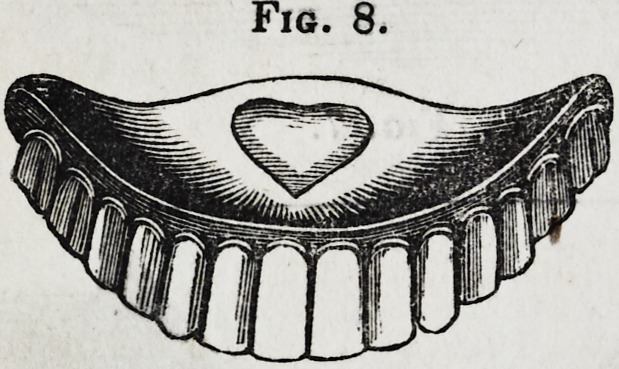# Single Porcelain Teeth Mounted on a Socket Plate

**Published:** 1853-10

**Authors:** Chapin A. Harris


### ARTICLE XI.

*Single Porcelain Teeth Mounted on a Socket Plate.* By Chapin A. Harris, M. D., D. D. S.

The liability of the platina pins in single porcelain teeth to break or be drawn from the teeth, is well known to almost every dentist. The frequency of its occurrence is often a source not only of serious inconvenience and great annoyance to persons wearing dental substitutes composed of single porcelain teeth mounted on a plate, but also of much trouble and vexation to the dentist; for oftentimes, in replacing a single tooth, when it becomes necessary to do it with solder, several others are cracked or broken. Besides, in heating a piece after it has been worn, there is always danger of springing the plate and destroying the accuracy of its adaptation to the mouth—an accident, which, in some cases, can only be remedied by reconstructing the entire apparatus.

The best translucent porcelain teeth that are made when mounted on plate in the usual manner, sometimes break or have the platina pins drawn, owing to the vascillation to which they are subjected in biting and mastication; and even when the pins do not loosen or give way, the backings, connecting the teeth to the plate to which they are attached, sometimes break. The force applied to artificial teeth in the mouths of some persons is so great, that it is impossible to prevent them from being shaken more or less, when not held in place by some other means than the backings which connect them to the plate.

To obviate these difficulties, Dr. S. P. Hullihen, of Wheeling, Va., about seventeen years ago, contrived a kind of socket plate, by which the end of each tooth resting upon the base, fits into a sort of chamber. Teeth mounted in this way cannot be made to move perceptibly by any amount of force ordinarily applied to a dental substitute. It requires more labor, however, to construct a set thus, than in the usual way, but when completed, the piece is, unquestionably, worth more than double to the wearer.

Fig. 1.

Fig. 2.

In mounting teeth upon a socket plate, the following is, I believe, the method of procedure adopted by Dr. Hullihen: The impression of the mouth, plaster model and metallic castings are obtained in the usual manner. The plate is then swaged, the teeth selected, arranged, fitted, and retained in place by means of a rim of yellow wax. They are, at the same time, properly antagonized. The piece now presents the appearance as shown in fig. 1. This done, a rim of softened wax is applied with sufficient force to the outer surface of the teeth and margin of the plate, to obtain a perfect impression. In this part of the operation considerable care is necessary to prevent altering the adjustment of the teeth, and when pressed with sufficient force, the edges and outer surface of the wax is trimmed and a band of thin tin, of proper width, with serrated edges is applied. The wax may now be removed without altering its relation to the teeth and plate. From the impression thus procured, as seen in fig. 2, a plaster model, as shown in fig. 3, is obtained.

Fig. 3.

An impression is made with this in sand, but the withdrawal of the model, owing to its peculiar shape, is often attended with difficulty, unless sectional moulding flasks are used. Having accomplished this, the metallic castings are easily made.

Fig. 4.

Fig. 6.

Fig. 5.

Fig. 7.

Between the castings, a thin, narrow plate is struck up, to fit around the upper part of the teeth and outer exposed margin of the base. This, separate from the teeth and plate, is represented in fig. 4, and when applied, in fig. 6. The portions of this band which embrace the outer surface of the ends of the teeth in contact with the plate, are now filed, with an oval or half round file, until this part presents a scolloped or festooned appearance, as seen in fig. 5. It is represented in fig. 7 as applied to the teeth and plate. Thus prepared and fitted, it is held in place with wax properly applied. When this has hardened, the teeth and wax to which they have hitherto been adjusted, are removed. A thick paste of plaster of paris is now applied for the purpose of uniting the narrow festooned strip of plate and the base. When this has hardened and become perfectly dry, the wax is removed, borax and solder applied along the point of union on the edges of the two plates, which are united by flowing the solder with heat from the flame of a lamp. The teeth are readjusted, backings put on, and the other parts of the operation gone through with in the usual way. The narrow strip passing around the ends of the teeth, resting on the base, should be long enough to pass behind the last tooth on each side, so that in soldering, it may be united to the backing. This strip serves the same purpose to single, that the outer rim does to block teeth. The principle is similar to the one recommended by the writer in the fifth edition of his "Principles and Practice of Dental Surgery," of placing a co-let around the upper end of each tooth.

Fig. 8.

Teeth mounted in the manner as just described, when the work is properly executed, present a most beautiful appearance. A set for the upper jaw is represented in fig. 8. Apart from the advantages already mentioned, which they possess, over teeth put up in the usual way, the chamber or socket, into which the plate end of each tooth fits, prevents particles of food and other foreign matter from getting between the teeth and plate. There are some cases, it is true, in which it is necessary to cover the outer margin of the plate with the upper ends of the teeth, to prevent exposing it when the upper lip is raised. It would not, of course, be proper, in a mouth where this would occur, to use a socket plate.

## Figures and Tables

**Fig. 1. f1:**
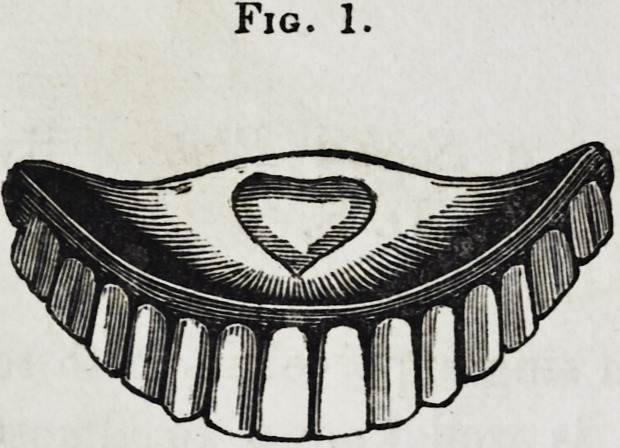


**Fig. 2. f2:**
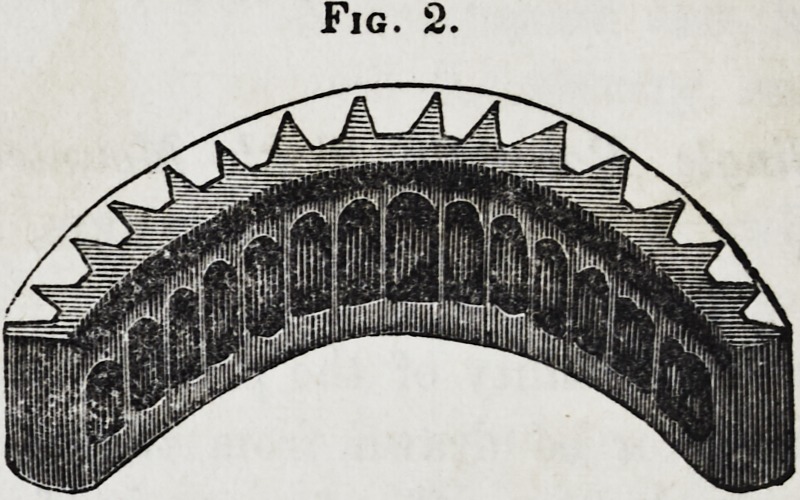


**Fig. 3. f3:**
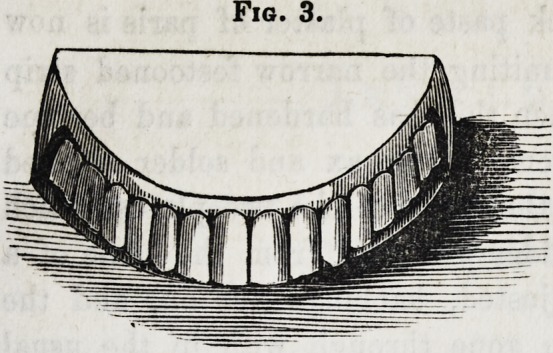


**Fig. 4. f4:**
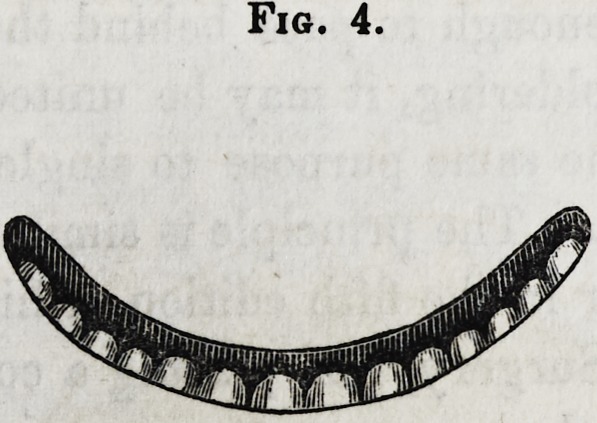


**Fig. 5. f5:**
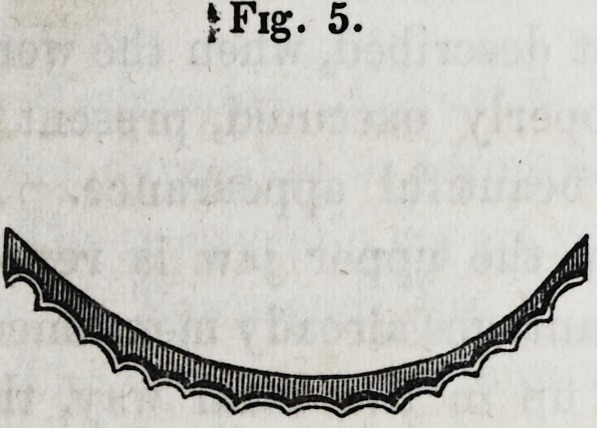


**Fig. 6. f6:**
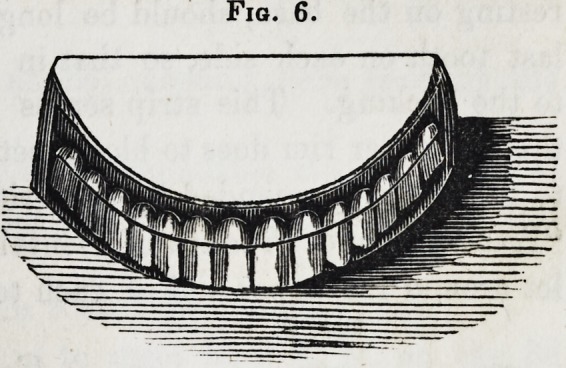


**Fig. 7. f7:**
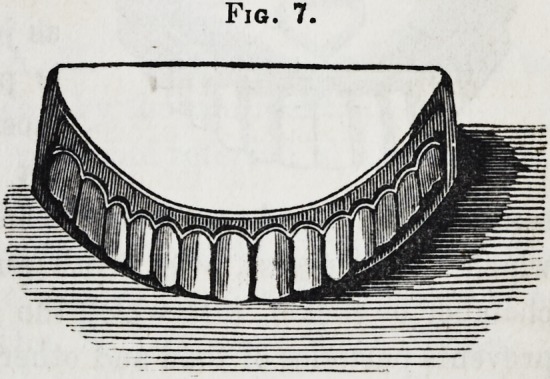


**Fig. 8. f8:**